# Exploring the gut mycobiome: differential composition and clinical associations in hypertension, chronic kidney disease, and their comorbidity

**DOI:** 10.3389/fimmu.2023.1317809

**Published:** 2023-12-14

**Authors:** Juan Qiu, Longyou Zhao, Yiwen Cheng, Qiaoxia Chen, Yiran Xu, Yingfeng Lu, Jie Gao, Wenhui Lei, Chengmin Yan, Zongxin Ling, Shaochang Wu

**Affiliations:** ^1^ Prenatal Diagnosis Center, Longhua Maternity and Child Healthcare Hospital, Shenzhen, Guangdong, China; ^2^ Department of Laboratory Medicine, Lishui Second People’s Hospital, Lishui, Zhejiang, China; ^3^ Collaborative Innovation Center for Diagnosis and Treatment of Infectious Diseases, State Key Laboratory for Diagnosis and Treatment of Infectious Diseases, National Clinical Research Center for Infectious Diseases, the First Affiliated Hospital, School of Medicine, Zhejiang University, Hangzhou, Zhejiang, China; ^4^ Jinan Microecological Biomedicine Shandong Laboratory, Jinan, Shandong, China; ^5^ Department of Laboratory Medicine, Shandong First Medical University, Jinan, Shandong, China; ^6^ Department of Intensive Unit, Hangzhou Jiaye Rehabilitation Hospital, Hangzhou, Zhejing, China

**Keywords:** gut mycobiome, hypertension, chronic kidney disease, cytokine, renal function

## Abstract

**Background:**

Hypertension (HTN) and chronic kidney disease (CKD) pose significant global health challenges and often coexist, amplifying cardiovascular risks. Recent attention has turned to the gut mycobiome as a potential factor in their pathophysiology. Our study sought to examine the gut fungal profile in individuals with HTN, CKD, and the concurrent HTN+CKD condition, investigating its connections with serum cytokines, renal function, and blood pressure.

**Methods and materials:**

We investigated three distinct participant groups: a cohort of 50 healthy controls (HC), 50 individuals diagnosed with HTN-only, and 50 participants suffering from both HTN and CKD (HTN+CKD). To facilitate our research, we gathered fecal and blood samples and conducted a comprehensive analysis of serum cytokines. Moreover, fungal DNA extraction was conducted with meticulous care, followed by sequencing of the Internal Transcribed Spacer (ITS) region.

**Results:**

HTN+CKD patients displayed distinctive fungal composition with increased richness and diversity compared to controls. In contrast, HTN-only patients exhibited minimal fungal differences. Specific fungal genera were notably altered in HTN+CKD patients, characterized by increased *Apiotrichum* and *Saccharomyces* levels and reduced *Candida* abundance. Our correlation analyses revealed significant associations between fungal genera and serum cytokines. Moreover, certain fungal taxa, such as *Apiotrichum* and *Saccharomyces*, exhibited positive correlations with renal function, while others, including *Septoria*, *Nakaseomyces*, and *Saccharomyces*, were linked to blood pressure, particularly diastolic pressure.

**Conclusion:**

Gut mycobiome dysbiosis in individuals with comorbid HTN and CKD differs significantly from that observed in HTN-only and healthy controls. The interactions between serum cytokines, renal function, and blood pressure emphasize the potential impact of the fungal microbiome on these conditions. Additional research is required to clarify the underlying mechanisms and identify therapeutic opportunities associated with mycobiome dysbiosis in HTN and CKD.

## Introduction

Hypertension (HTN) is a widespread cardiovascular condition of global significance, standing as a primary risk factor for various cardiovascular diseases such as stroke, coronary artery disease, and heart failure. Consequently, it significantly contributes to global morbidity and mortality (1). Concurrently, chronic kidney disease (CKD) has become an escalating health challenge, impacting millions worldwide and frequently coexisting with HTN (2). The convergence of HTN and CKD introduces a compounding effect on cardiovascular risk, rendering this comorbidity a focal point of intense research interest (3).

The intricate relationship between HTN and CKD is marked by bidirectional interactions. HTN can contribute to the initiation and progression of CKD, while CKD, in turn, can exacerbate HTN (4). Although conventional risk factors like age, gender, genetics, and lifestyle play crucial roles in the development of HTN and CKD (5), recent findings underscore the impact of non-traditional factors. Notably, the gut microbiome has gained attention (5), with a specific focus on the mycobiome—the fungal component of the microbiome—in shaping the pathophysiology of these conditions (6, 7).

Recent advances in high-throughput sequencing technologies have facilitated the comprehensive profiling of fungal communities within the human body. This has shed light on their intricate roles in maintaining physiological equilibrium and influencing disease states. Particularly, the gut mycobiome has emerged as a significant player in various gastrointestinal disorders, including inflammatory bowel disease and colorectal cancer, highlighting the relevance of fungi in human health (8–10), which extends to hypertension and kidney disease. For example, a study by Zou et al. revealed associations between changes in the composition of the gut mycobiome and hypertension (7), suggesting a potential role of fungi in blood pressure regulation. Additionally, research by Hu et al. documented substantial differences in the fungal community structure between chronic kidney disease (CKD) patients and healthy controls (11), implicating mycobiome dysbiosis in the context of renal dysfunction.

Given the intricate interplay between the gut microbiome, including the mycobiome, and systemic physiology, it is plausible that mycobiome dysbiosis, characterized by shifts in fungal composition and diversity, may play a substantive role in the pathogenesis of hypertension (HTN) and chronic kidney disease (CKD). A comprehensive understanding of how mycobiome dysbiosis interfaces with immune responses, renal function, and blood pressure regulation in the context of HTN and CKD is imperative for unveiling novel mechanistic insights and identifying prospective therapeutic targets. In consideration of this, our study seeks to explore the complex interconnections between fungal dysbiosis, serum cytokine profiles, renal function, and blood pressure levels in individuals with HTN, specifically focusing on the overlapping HTN and CKD (HTN+CKD) phenotype.

## Materials and methods

### Study cohort and participants recruitment

Three distinct participant groups were recruited for the study: healthy controls, individuals with hypertension only (HTN-only), and individuals with hypertension accompanied by chronic kidney disease (HTN+CKD). For the HTN-only group, a total of 50 subjects were recruited, evenly divided between 25 females and 25 males. Hypertension was confirmed using the following criteria: systolic blood pressure (SBP) ≥140 mmHg or diastolic blood pressure (DBP) ≥90 mmHg, as determined through repeated examinations (12), or based on the use of antihypertensive medication. Blood pressure measurements were conducted with participants seated, administered by trained nurses. Three readings were taken at 5-minute intervals using a random-zero mercury column sphygmomanometer, with the recorded average value considered the final measurement. The HTN+CKD group comprised 50 non-dialyzed patients with both hypertension and CKD (25 females and 25 males). CKD was defined as the presence of renal structural abnormalities and dysfunction persisting for more than 3 months, supported by at least one renal injury marker and/or an estimated glomerular filtration rate (eGFR) < 60 mL/min·1.73m^2^ (13, 14). The healthy control group consisted of 50 participants (25 females and 25 males) who had no history of hypertension, kidney damage, or kidney function loss. Age-matching was ensured across all three groups of participants.

Study participants were ineligible if they fulfilled any of the subsequent exclusion criteria: acute intercurrent diseases and infections, cancer, stroke, peripheral artery disease, heart failure, renal failure, kidney damage, diabetes, pregnancy, breastfeeding, elevated body temperature, or white blood cell count, as well as recent use of antibiotics, probiotics, or immunosuppressive drugs within 60 days preceding enrollment.

This study received approval from the ethics committee of Lishui Second People’s Hospital (20230119-01). All subjects provided informed consent before their inclusion in the study.

### Sample collection and processing

Participants were provided with instructions for the collection and processing of fecal samples. They were instructed to gather fecal samples in sterile containers and transfer approximately 30mg of feces into a sterile bottle containing 1000μL of lysis buffer. This lysis buffer comprised Tris 0.1mol/L (pH 8.0), 2 mM EDTA, and 2% SDS (Guhe Health, Hangzhou, China). Post-collection, these samples were promptly stored at -80°C until further processing.

Simultaneously, alongside the collection of fecal samples, blood samples were obtained for the assessment of renal function and the analysis of serum cytokines. The Bio-Plex™ 200 System, manufactured by Bio-Rad, and the Bio-Plex Pro™ Human Cytokine Screening 48-plex Panel, also from Bio-Rad in California, USA, were utilized for the detection of serum cytokines.

### Fungal DNA extraction

The isolation of fungal genomic DNA from fecal samples utilized the DNeasy PowerSoil Pro Kit following the manufacturer’s instructions, conducted within a biological safety cabinet (QIAGEN, Germany). To enhance the process, additional glass-bead beating steps were incorporated using a Mini-Beadbeater (FastPrep; Thermo Electron, Boston, MA, United States). The quantification of DNA content was determined with a NanoDrop ND-1000 spectrophotometer (Thermo Electron). Subsequently, the integrity and size of the DNA were verified through electrophoresis on a 1.0% agarose gel containing 0.5 mg/ml ethidium bromide. All DNA samples were then stored at -20°C until further analysis.

### ITS region amplification

The amplification of ITS regions was achieved using the ITS1F (5’-CTTGGTCATTTAGAGGAAGTAA-3’) and ITS2 (2043R; 5’-GCTGCGTTCTTCATCGATGC-3’) primers. PCR reactions were performed with Phusion High-Fidelity PCR Master Mix (Thermo Scientific Inc., Waltham, MA, USA) following the manufacturer’s protocol, employing approximately 50 ng of extracted DNA per reaction. The thermocycling conditions included an initial denaturation at 98°C for 15 sec in one cycle, followed by 30 cycles of denaturation at 98°C for 15 sec, annealing at 58°C for 15 sec, extension at 72°C for 15 sec, and a final extension at 72°C for 60 sec.

Negative DNA extraction samples, composed of lysis buffer and kit reagents only, were subjected to amplification and sequencing to function as contamination controls. Amplified products underwent purification using Agencourt AMPure XP beads (1 volume; Beckman Coulter, Pasadena, CA), and the samples were subjected to gel electrophoresis for size selection, targeting gel slices of approximately 430 bp in size. DNA quantification was performed using a Qubit 2.0 Fluorometer (Life Technologies, Carlsbad, California, US). Sequencing was conducted with 2×150bp sequencing on a Novaseq 6000 platform (Illumina Inc., San Diego, CA, USA).

### Bioinformatic analysis

#### ITS sequence data processing

The ITS sequence dataset underwent merging and demultiplexing for the acquisition of per-sample data, employing QIIME (V1.9.1) with default parameters (15). Raw sequencing reads were attributed to their respective samples through exact matches to the barcodes, thereby identifying valid sequences. Subsequently, low-quality sequences were filtered based on specific criteria, including a length of <150bp, an average Phred score of <20, the presence of ambiguous bases, and mononucleotide repeats exceeding 8 bp. In the case of paired-end reads, assembly was conducted using Vsearch (V2.4.4; -fastq_mergepairs-fastq_minovlen 6).

Operational taxonomic unit (OTU) picking involved several steps, including dereplication (-derep_full length), clustering (-cluster_fast, -id 0.97), and chimera detection (-uchime_ref). From each OTU, a representative sequence was chosen using default parameters. Taxonomic classification of OTUs was achieved by searching the representative sequences set against the UNITE 12_11 database (https://unite.ut.ee) using Vsearch (16).

#### OTU table generation and abundance filtering

An OTU table was generated to record the abundance of each OTU in every sample along with the taxonomy of the OTUs. To facilitate unbiased analyses and normalize the OTU table, a minimum library size was selected for rarefying the OTUs in our study. Total sum scaling (TSS) was employed to convert the OTU table into relative abundance by dividing the total reads of each sample. OTUs constituting less than 0.01% of the total sequences across all samples were excluded.

### Sequence data analysis

The analysis of sequence data was conducted using QIIME and R package (V3.2.0). Alpha richness and diversity indices at the OTU level, such as Chao 1, Shannon, and Simpson, were computed using the OTU table. For beta diversity analysis, investigating the structural variation of fungal communities across samples, Bray-Curtis metrics were utilized. The results were visualized through principal coordinate analysis (PCoA) based on permutational multivariate analysis of variance (PERMANOVA) calculated using the Adonis function (17–19).

### Statistical analysis

Categorical variables underwent analysis using Pearson’s Chi-square or Fisher’s exact tests, while normalized continuous variables were subjected to Student’s t-test and ANOVA. In the case of non-normally distributed continuous variables, the Wilcoxon rank-sum test was applied. To address the potential impact of multiple comparisons, P-values were adjusted using the Benjamini–Hochberg (BH) false discovery rate (FDR) correction.

To investigate the associations between mycobiome dysbiosis and serum cytokines, we initially evaluated the concentrations of serum cytokines. Subsequently, we conducted Pearson correlation analyses to explore the correlations between the genera showing differences in the HTN+CKD group compared to the other two groups and cytokines. Additionally, we performed Pearson correlation analyses to examine the relationships between the modified fungal genera and the eGFR and blood pressure of the patients.

## Results

### Participant characteristics

In this study, three participant groups were recruited: 50 healthy controls (HC), 50 individuals with hypertension (HTN-only), and 50 subjects with both hypertension and chronic kidney disease (HTN+CKD). **Table 1** presents the demographic characteristics of these groups. Our study findings reveal that there were no statistically significant differences in age, gender distribution, body mass index (BMI), smoking history, or drinking history among the three groups (P>0.05). Similarly, no significant disparities were observed in the duration of hypertension or the use of antihypertensive medications between the HTN-only and HTN+CKD groups (P>0.05). As expected, participants in the HTN-only and HTN+CKD groups exhibited significantly higher levels of systolic and diastolic blood pressure compared to the control group (P<0.05). Additionally, the HTN+CKD group demonstrated a significant decline in estimated glomerular filtration rate (eGFR) and notably higher levels of serum creatinine, blood urea nitrogen, and serum uric acid compared to the control group (P<0.05).

**Table 1:** Demographics of fecal donors.

Characteristics	HC (n=50)	HTN-only (n=50)	HTN+CKD (n=50)	*P-*value
Demographics				
Age-years	57.10 ± 14.09	56.11 ± 17.94	57.44 ± 16.43	0.931
Male sex-no.(%)	25 (50.00)	25 (50.00)	25 (50.00)	1.000
Body mass index-kg/m^2^	24.14 ± 3.06	24.26 ± 4.47	23.73 ± 3.51	0.687
Smoking history-no.(%)				0.055
Never smoker	46 (92.00)	42 (84.00)	43 (86.00)	
Former smoker	2 (4.00)	4 (8.00)	6 (12.00)	
Current smoker	2 (4.00)	4 (8.00)	1 (2.00)	
Drinking history-no. (%)				0.068
Never drinker	46 (92.00)	44 (88.00)	45 (90.00)	
Former drinker	2 (4.00)	4 (8.00)	3 (6.00)	
Current drinker	2 (4.00)	2 (4.00)	2 (4.00)	
Duration of HTN-years	NA	11.12 ± 8.87	12.00 ± 5.74	0.910
Duration of CKD-years	NA	NA	6.99 ± 4.60	NA
Systolic blood pressure-mmHg	128.30 ± 16.88	129.78 ± 17.17	147.37 ± 14.70	<0.001
Diastolic blood pressure-mmHg	94.42 ± 3.52	81.04 ± 7.52	91.93 ± 17.53	<0.001
Renal function				
eGFR-mL/min/1.73m^2^	102.22 ± 12.18	101.86 ± 16.76	38.84 ± 21.97	<0.001
CKD stage-no. (%)				NA
Stage 3	NA	NA	15 (30.00)	
Stage 4	NA	NA	15 (30.00)	
Stage 5	NA	NA	20 (40.00)	
24h urinary protein-mg/dL	NA	NA	5108.17 ± 2641.14	NA
Serum creatinine-μmol/L	66.68 ± 21.47	139.32 ± 11.26	259.39 ± 218.61	<0.001
Blood urea nitrogen-mmol/L	5.26 ± 2.67	7.55 ± 5.38	19.53 ± 9.24	<0.001
Serum uric acid-umol/L	298.77 ± 104.12	324.37 ± 109.89	436.33 ± 189.28	<0.001
24h urine output-mL	2409.17 ± 239.18	2181.58 ± 369.38	1347.94 ± 311.68	<0.001
Microalbuminuria ≥200mg/L-no. (%)	0	0	45 (90.00)	<0.001
Antihypertensive agent	NA	35(70.00)	38 (76.00)	0.499

CKD, chronic kidney disease; HTN, hypertension.

Pearson Chi-square or Fisher’s exact test was used with categorical variables; Student’s t test on normalized continuous variables was used.NA: not applicable.

### Distinct fungal profiles in hypertension with chronic kidney disease patients

**Figure 1:**
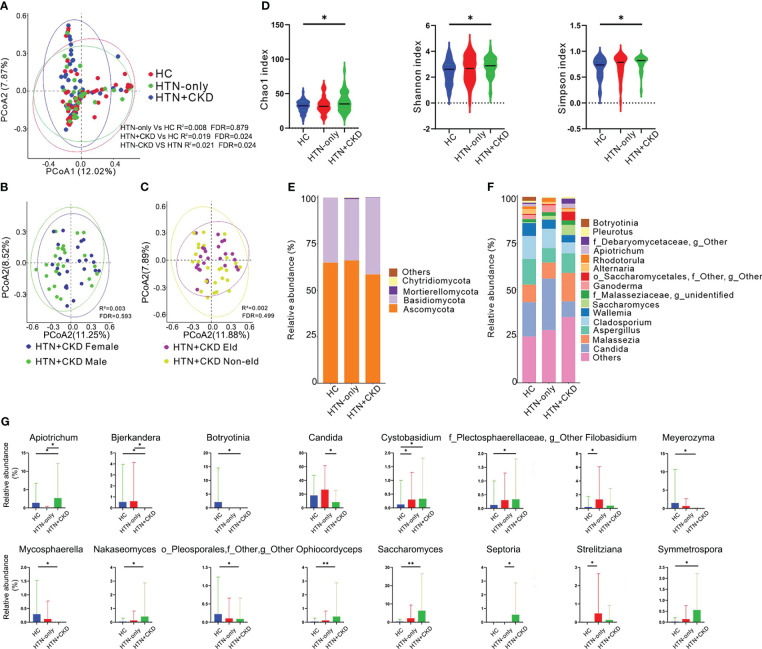
Mycobiome Composition, Diversity, Phylum and Genus Profiles, and Inter-Group Comparisons. **(A-C)** Principal Coordinate Analysis (PCoA) based on Bray-Curtis distances at the Operational Taxonomic Unit (OTU) level, illustrating the microbial community comparisons among healthy controls (HC), hypertension (HTN-only), and hypertension with chronic kidney disease (HTN+CKD) groups, among HTN+CKD female and HTN+CKD male, and among HTN+CKD eld and HTN+CKD non-eld. A 95% confidence ellipse is plotted for each group. Permutational multivariate analysis of variance (PERMANOVA) was applied for statistical comparisons between the groups, with *P*-values adjusted using the Benjamini and Hochberg false discovery rate (FDR) correction. **(D)** Assessment of fungal richness and diversity using Chao 1, Shannon, and Simpson indices at the OTU level. Wilcoxon rank-sum tests were employed, and *P*-values were adjusted for multiple comparisons using the Benjamini and Hochberg FDR correction. **(E, F)** Fungal taxonomic profiles at the phylum and genus levels, with representation limited to the top 15 most abundant genera. **(G)** Comparative analysis of fungal genera across the HC, HTN-only, and HTN+CKD groups. Wilcoxon rank-sum tests were employed, and *P*-values were adjusted for multiple comparisons using the Benjamini and Hochberg FDR correction. *, FDR < 0.05; **, FDR<0.01 CKD, chronic kidney disease; HC, healthy controls; HTN, hypertension.

As depicted in **Figure 1A**, Principal Coordinates Analysis (PCoA) revealed distinct fungal compositions in HTN+CKD patients compared to both HTN-only patients and controls (FDR<0.05) (17–19). Importantly, HTN-only patients exhibited no significant differences in fungal composition compared to controls (FDR=0.879). To assess whether the differences between the HTN+CKD group and the HTN-only and control groups were influenced by gender and age, we further subdivided the HTN+CKD patients into different gender and age groups. We observed no significant differences between male and female subgroups (FDR<0.05; **Figure 1B**) and between elderly and non-elderly subgroups (FDR<0.05; **Figure 1C**).

When examining fungal richness, the HTN+CKD group exhibited a higher Chao 1 level than the control group (**Figure 1D**; FDR<0.05). Similarly, HTN+CKD patients demonstrated elevated levels of fungal diversity compared to controls, as indicated by both the Shannon index and Simpson index (**Figure 1D**; FDR<0.05).

At the fungal phylum level (**Figure 1E**), all three groups were primarily dominated by *Ascomycota* (comprising 60.17%, 60.18%, and 51.59% in the HC, HTN-only, and HTN+CKD groups, respectively) and *Basidiomycota* (comprising 32.21%, 30.13%, and 36.38% in the HC, HTN-only, and HTN+CKD groups, respectively).

Examining fungal genera (**Figure 1F** and **Supplementary Figure S1**), the HC group featured dominant genera such as *Candida* (20.30%), *Aspergillus* (15.25%), *Cladosporium* (13.49%), *Malassezia* (10.37%), and *Wallemia* (7.77%). The HTN-only group was marked by dominant genera, including *Candida* (30.37%), *Cladosporium* (12.08%), *Malassezia* (10.26%), *Aspergillus* (8.11%), and *Wallemia* (5.70%). In contrast, the HTN+CKD group displayed dominance by *Malassezia* (18.71%), *Aspergillus* (12.93%), *Candida* (9.76%), *Saccharomyces* (7.38%), and *Cladosporium* (6.67%).

When comparing fungal genera, a total of 13 genera were identified with significantly different abundances between the HTN+CKD group and the control group. Among these, 8 genera exhibited increased abundances in the HTN+CKD group, while 5 genera displayed decreased abundances (FDR<0.05; **Figure 1G** and **Supplementary Figure S2**). Notably, the HTN+CKD group demonstrated higher abundances of genera such as *Apiotrichum*, *Cystobasidium*, *Saccharomyces*, and more, while showing lower abundances of genera like *Bjerkandera*, *Candida*, *Meyerozyma*, among others (FDR<0.05; **Figure 1G**). In comparison to the control group, only 3 genera exhibited significantly different abundances in the HTN-only group, with higher abundances of *Cystobasidium*, *Filobasidium*, and *Strelitziana* (FDR<0.05; **Figure 1G**). Additionally, when compared to the HTN-only group, the HTN+CKD group displayed higher abundances of *Apiotrichum* and *Septoria*, while exhibiting lower abundances of *Candida* (FDR<0.05; **Figure 1G**).

### Mycobiome dysbiosis links to serum cytokines, renal function, and blood pressure

**Figure 2:**
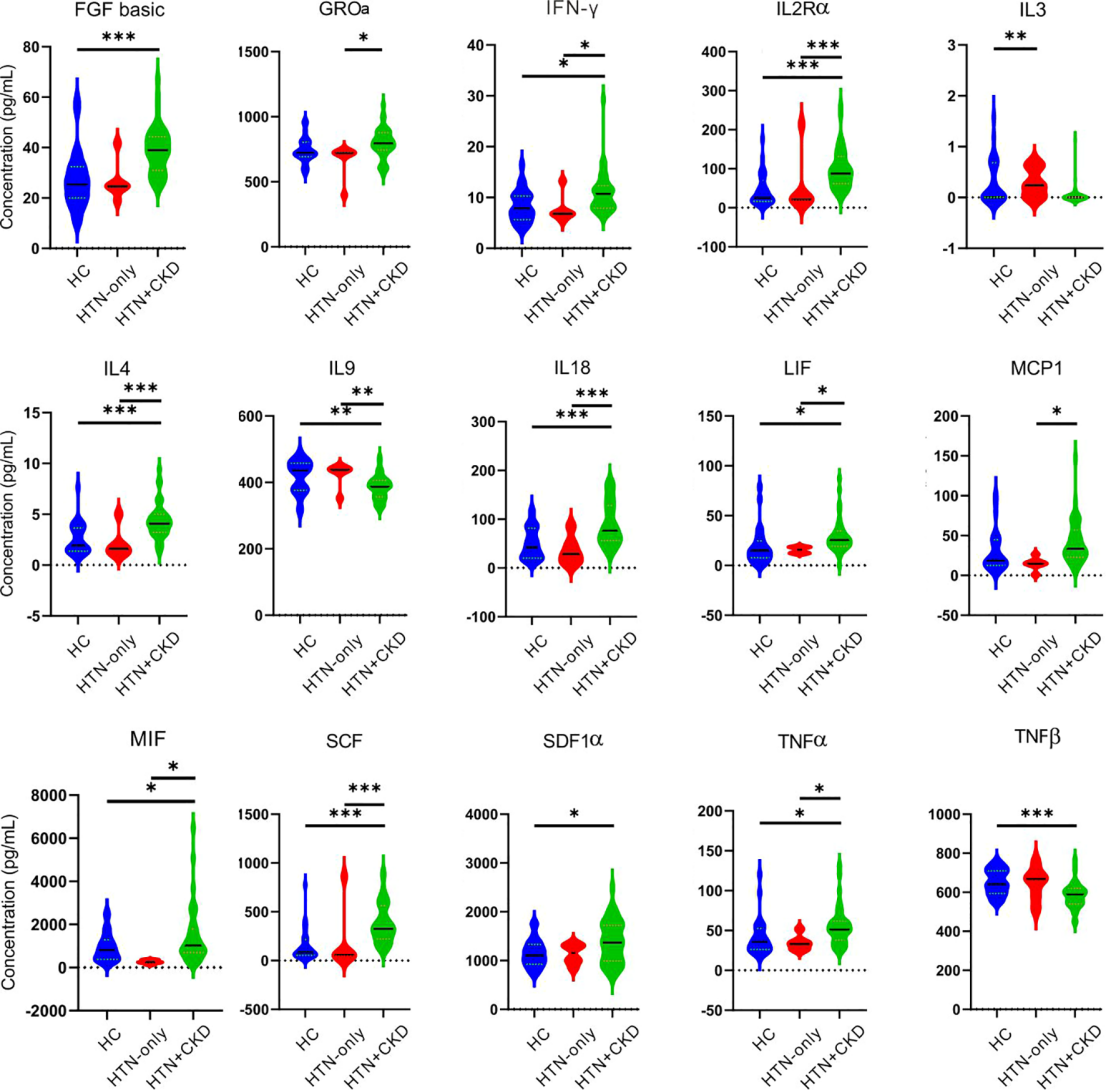
Comparative analysis of serum cytokines across the HC, HTN-only, and HTN+CKD groups. Student’s *t* test and ANOVA were used on normalized continuous variables and Wilcoxon rank-sum test on non-normal continuous variables. The *P*-value was adjusted for multiple comparisons using the Benjamini–Hochberg (BH) false discovery rate (FDR). *, *P*/FDR < 0.05; **, *P*/FDR<0.01; ***P/FDR<0.001. CKD, chronic kidney disease; HC, healthy controls; HTN, hypertension.

We observed significant differences in the concentrations of 12 cytokines between the HTN+CKD group and the control group. Notably, HTN+CKD patients had higher concentrations of IL2Rα, IL18, and TNFα compared to the controls, while exhibiting lower concentrations of IL9 and TNFβ (*P*<0.05; **Figure 2**). Additionally, 11 cytokines displayed significantly different concentrations between the HTN+CKD group and HTN-only patients, with the HTN+CKD group showing higher concentrations of IL2Rα, IL18, and TNFα and lower concentrations of IL9 (*P*<0.05; **Figure 2**). Interestingly, only one cytokine, IL3, exhibited a different concentration between the HTN-only patients and controls (*P*<0.05; **Figure 2**).

**Figure 3:**
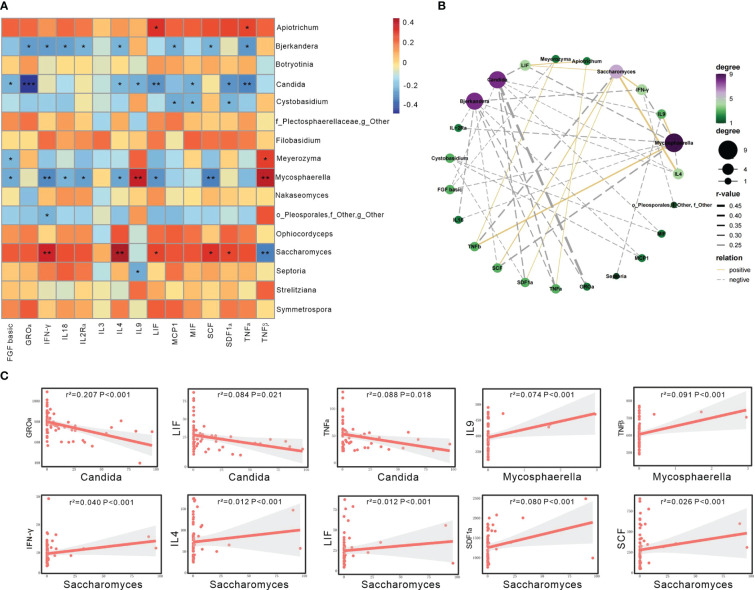
Gut mycobiome was associated with participants’ serum cytokines. **(A)** The heatmap depicted the association between the fungal genera and serum cytokines showing differed among the three groups of HC, HTN-only and HTN+CKD. Spearson correlation analysis was performed. The correlation of two variables with values of |*r*|>0.3 and *P < *0.05 are displayed. *, *P < *0.05; and **, *P < *0.01. **(B)** Network analysis of the correlation between abnormal genera and discriminatory cytokines. Yellow line represents positive correlations and gray negative correlations. **(C)** Linear regression analysis was conducted to examine the relationship between abnormal genera and discriminatory cytokines.

Our observations revealed that *Candida* showed negative associations with several serum cytokines, including GROα, LIF, and TNFα (*P*<0.05; **Figures 3A, B**). *Mycosphaerella* exhibited negative correlations with several serum cytokines, such as FGF basic, IFN-γ, IL18, IL2Rα, IL4, LIF, SCF, while demonstrating positive correlations with IL9 and TNFβ (*P*<0.05). *Saccharomyces* displayed positive correlations with several serum cytokines, including IFN-γ, IL4, LIF, SCF, and SDF1ɑ, while having a negative correlation with TNFβ (*P*<0.05; **Figures 3A, B**).

The linear regression analysis revealed that several of the aforementioned taxa can account for the changes in serum cytokines in patients with HTN+CKD. Specifically, Candida exhibited a negative association with GROɑ, LIF, and TNFɑ (P<0.05; **Figure 3C**), while Saccharomyces showed a positive association with IFN-γ, IL4, LIF, SCF, and SDF1ɑ (P<0.05; **Figure 3C**).

**Figure 4:**
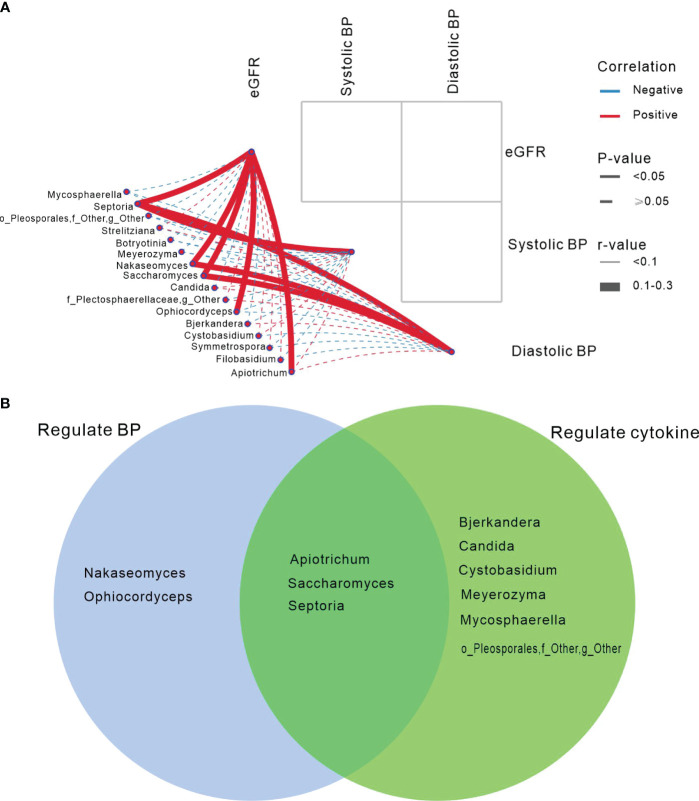
Gut mycobiome was associated with participants’ eGFR/blood pressure, and cytokines. **(A)** The correlation analysis depicted the association between the fungal genera and eGFR/blood pressure showing differed in among the three groups of HC, HTN-only and HTN+CKD. Spearson correlation analysis was performed. Solid lines represent statistically significant correlations (*P*<0.05); dashed lines indicate no statistically significant correlations (*P*≥0.05). Red lines denote positive correlations, while blue lines indicate negative correlations. **(B)** Abnormal genera regulated BP and cytokines.

We found that *Apiotrichum*, *Ophiocordyceps*, *Saccharomyces*, *Nakaseomyces*, and *Septoria* exhibited positive associations with eGFR (*P*<0.05). *Septoria* was positively correlated with systolic and diastolic blood pressure, while *Nakaseomyces* and *Saccharomyces* were positively associated with diastolic blood pressure (*P*<0.05; **Figure 4A**).

Venn demonstrated that *Apiotrichum*, *Saccharomyces*, and *Septoria* not only regulate blood pressure but also modulate blood cytokines. However, *Nakaseomyces* and *Ophiocordyceps* only influence blood pressure, while *Bjerkandera*, *Candida*, and *Cystobasidium* exclusively regulate cytokines (**Figure 4B**).

## Discussion

In our study, we have embarked on a pioneering investigation into the fungal microbiome of individuals with comorbid hypertension and CKD, shedding light on the intricate connections between mycobiome dysbiosis and various facets of health and disease. While previous research has identified disruptions in the fungal microbiome within the intestines of individuals with hypertension or CKD (7, 20), there has been a notable absence of studies examining the gut fungal microbiome in individuals concurrently afflicted with both conditions.

Hypertension, a well-established risk factor for renal dysfunction development (2), provides a unique opportunity to explore the potential interplay between mycobiome dysbiosis, immune responses, renal function, and blood pressure regulation. Our findings unveil distinctive characteristics in the fungal microbiome of individuals with both hypertension and CKD compared to those with hypertension alone, without kidney function impairment, as well as healthy control groups. The significant variations observed in gut mycobiome composition among individuals with comorbid hypertension and CKD highlight the potential impact of CKD in shaping mycobiome dysbiosis. However, it is crucial to acknowledge that differences among these three groups may be influenced, to some extent, by oral medications rather than the diseases themselves. This factor should be considered in our interpretation of the findings.

In contrast, individuals with hypertension alone did not exhibit substantial differences in fungal diversity compared to the control group. The heightened fungal richness and diversity observed in individuals with both hypertension and CKD suggest alterations in the gut mycobiome, potentially correlated with hypertension and CKD. This observation aligns with prior research emphasizing the association of mycobiome dysbiosis with various diseases (8, 21, 22). However, it is essential to refrain from implying a causal relationship between the flora and diseases, as our study does not provide insight into the underlying reasons.

While studies on bacterial gut microbial ecology have consistently shown lower diversity in patients compared to healthy individuals, the landscape differs in the realm of fungal microbiota research. At times, patients exhibit higher microbial diversity than their healthy counterparts. For instance, Nelson et al. identified a significant increase in observed operational taxonomic units (OTUs) in the gut mycobiota of Crohn’s disease patients in remission compared to healthy individuals, accompanied by a slight rise in the Shannon index (23). Shah et al. observed significantly higher alpha diversity in patients with multiple sclerosis compared to healthy controls (24). In another study, Lemoinne et al. found that patients with sclerosing cholangitis, either alone or in combination with inflammatory bowel disease, demonstrated significantly higher bacterial diversity than healthy controls (25).

Our examination of fungal genera abundance in this study revealed compelling insights into the mycobiome dysbiosis associated with comorbid hypertension and CKD. Notable alterations in fungal composition, particularly the increased abundance of genera such as *Apiotrichum*, *Cystobasidium*, and *Saccharomyces*, in individuals with comorbid hypertension and CKD, shed light on the potential involvement of specific fungal taxa in the pathophysiology of these conditions. These genera have been previously implicated in various disease contexts (26–28), supporting the notion that they might play a significant role in the interplay between hypertension and CKD.

Conversely, the reduced abundance of *Candida* and potentially other fungal genera in individuals with both hypertension and CKD, compared to healthy controls, underscores the unique mycobiome dysbiosis profile associated with this comorbidity. *Candida*, a prevalent fungal genus, has been extensively studied and is known to have implications in various diseases, including gastrointestinal disorders and immunomodulation (29, 30). However, recent evidence indicates that the role of *Candida* in human health is complex. Commensal coexistence of *C. albicans* with the human host has likely conferred evolutionary benefits to human health, rather than being solely detrimental (31). Therefore, the lower abundance of *Candida* in individuals with comorbid hypertension and CKD observed in our study may merely indicate mycobiome dysbiosis in these patients, rather than implying an improvement in their overall health status.

Comparatively, individuals with hypertension alone displayed fewer alterations in fungal genera, highlighting a less pronounced mycobiome dysbiosis profile in this group. This finding aligns with existing research indicating that the gut mycobiome may undergo more profound changes in individuals with comorbidities or complex health conditions (32, 33). Understanding these differences in mycobiome dysbiosis profiles among various health states is critical for deciphering the specific role of the fungal microbiome in the pathogenesis of hypertension, CKD, and their comorbidity.

This study has identified significant inter-group differences in certain cytokines, particularly in the number of cytokines showing variations between the HTN+CKD group and the control group, with a greater disparity compared to the HTN-only group and the control group. Among these cytokines exhibiting inter-group differences, the majority have been previously reported in the literature to be associated with changes in kidney function, such as FGF basic (34), IL2Rɑ (35), IL4 (36), IL18 (37), MIF (38), SCF (39), SDF1ɑ (40), TNFɑ (41), and IL9 (42). The abnormal expression of these cytokines suggests that hypertensive patients, especially those with concurrent chronic kidney disease (CKD), induce alterations in the body’s immune function during the disease progression.

Our study explored the relationships between mycobiome dysbiosis and key parameters, including serum cytokine profiles, renal function, and blood pressure. Elevated concentrations of pro-inflammatory cytokines, including IFNγ and IL18 (43, 44), were notably observed in individuals with both hypertension and CKD. Conversely, lower concentrations of anti-inflammatory cytokines, such as IL9 (45), were evident, underscoring the potential involvement of chronic inflammation in these concurrent conditions. These findings align with prior research underscoring the significance of cytokines, particularly TNFα, in the pathogenesis of hypertension and kidney injury (46, 47).

Our correlation analyses provide additional insights into the potential immunomodulatory roles of specific fungal taxa. This study reveals a decrease in *Candida* levels in the HTN+CKD group, accompanied by an elevation in TNFα levels. Importantly, a negative correlation was observed between *Candida* and TNFα in this group. Similar phenomena have been reported in previous *in vitro* and *in vivo* experiments. For instance, Ohta et al.’s *in vitro* experiment found that exogenous TNFα can have either a beneficial or detrimental effect against *Candida*. Simultaneously, their *in vivo* experiment demonstrated that orally administered TNFα suppressed fungal burden in the tongue tissue (48).

The positive correlation between *Mycosphaerella* and TNFβ in HTN+CKD patients raises intriguing questions about the potential implications for disease progression. TNFβ is known to play a pivotal role in inflammation and immune response (49). Understanding whether *Mycosphaerella* actively contributes to TNFβ regulation or if the host’s response to the fungus triggers this pro-inflammatory cascade is essential for unraveling the mechanisms at play. Simultaneously, the positive association between *Mycosphaerella* and the anti-inflammatory cytokine IL9 introduces an intriguing dimension to the discussion. Given that HTN+CKD is characterized by chronic inflammation and immune dysregulation (50), the upregulation of IL9 in the presence of *Mycosphaerella* may represent a host response aimed at counteracting excessive inflammation. The positive correlations of *Mycosphaerella* with both TNFβ and IL9 in HTN+CKD patients suggest a potential immunoregulatory role for this fungal genus in the complex interplay between hypertension and chronic kidney disease. Further research is needed to decipher the specific mechanisms through which *Mycosphaerella* influences these cytokines and whether its presence exacerbates inflammation or triggers a compensatory anti-inflammatory response.

The observed positive association between *Saccharomyces* and crucial cytokines, namely IFN-γ, IL-4, LIF, SDF1α, and SCF, presents a compelling avenue for rational exploration into the immunomodulatory potential of this yeast. To be specific, the correlation with IFN-γ suggests a possible immunostimulatory effect of *Saccharomyces*. Considering the central role of IFN-γ in both innate and adaptive immune responses (51), the association with *Saccharomyces* raises questions about the yeast’s specific mechanisms for enhancing immune function. Secondly, the positive link with IL-4, known for its anti-inflammatory properties and modulation of Th2 responses (52, 53), hints at the potential of *Saccharomyces* to influence immune balance. This aspect could be particularly relevant in conditions characterized by excessive inflammation. Further research is warranted to elucidate whether *Saccharomyces* supplementation might offer a targeted approach to managing inflammatory disorders. The association with LIF, a cytokine involved in embryonic development and immune regulation (54), suggests that *Saccharomyces* could play a role in supporting immune homeostasis. Investigating the mechanisms behind this association may unveil novel therapeutic strategies for conditions influenced by immune dysregulation. SDF1α, a chemokine crucial for immune cell recruitment and homing (55), displays a positive correlation with Saccharomyces. This finding suggests that *Saccharomyces* might be involved in the regulation of immune cell trafficking. In conclusion, the integration of these findings underscores the need for further research to unravel the underlying mechanisms of the positive association between *Saccharomyces* and these key cytokines.

Moreover, our findings suggest intriguing associations between fungal genera and renal function and blood pressure regulation. The positive associations observed between fungal genera such as *Apiotrichum*, *Ophiocordyceps*, *Saccharomyces*, *Nakaseomyces*, and *Septoria* with estimated glomerular filtration rate (eGFR) hint at potential roles of these fungi in supporting or enhancing renal function. The correlations between fungal genera, particularly *Septoria*, *Nakaseomyces*, and *Saccharomyces*, with blood pressure, especially diastolic blood pressure, suggest a possible involvement of these fungi in blood pressure regulation. These observations align with emerging research suggesting a connection between the gut microbiome, including the mycobiome, and blood pressure regulation (7, 56).

One limitation of this study is its cross-sectional design, which restricts our ability to establish causal relationships. Longitudinal studies would be essential to determine the temporal dynamics of mycobiome dysbiosis in individuals with comorbid hypertension and CKD and how it relates to disease progression. Secondly, the sample size in this study consisted of 50 participants in each group. While this provided valuable insights, larger and more diverse cohorts could enhance the generalizability of the findings and uncover additional nuances in mycobiome dysbiosis associated with these conditions.

In conclusion, our study adds to the growing body of evidence highlighting the intricate interplay between the gut mycobiome and systemic physiology in the context of complex diseases. The distinct mycobiome dysbiosis profile associated with comorbid hypertension and CKD underscores the need for further research to elucidate the underlying mechanisms and therapeutic potential of targeting the mycobiome in the management of these conditions. Understanding the role of the mycobiome microbiome in hypertension and CKD may open new avenues for interventions aimed at modulating mycobiome dysbiosis and mitigating the impact of these comorbidities on health.

## Data availability statement

Raw data from ITS sequencing are available in the Sequence Read Archive under BioProject IDSRP12221476 (https://www.ncbi.nlm.nih.gov/sra/PRJNA895827).

## Ethics statement

The ethics committee of Lishui Second People’s Hospital approved this study (20230119-01). The studies were conducted in accordance with the local legislation and institutional requirements. The participants provided their written informed consent to participate in this study.

## Author contributions

JQ: Methodology, Project administration, Writing – original draft. LZ: Investigation, Software, Writing – original draft. YC: Software, Writing – original draft. QC: Data curation, Investigation, Methodology, Project administration, Software, Writing – original draft. YX: Investigation, Writing – original draft. YL: Investigation, Writing – review & editing. JG: Methodology, Writing – review & editing. WL: Investigation, Project administration, Writing – original draft. CY: Conceptualization, Supervision, Writing – original draft. ZL: Conceptualization, Formal analysis, Funding acquisition, Resources, Validation, Visualization, Writing – review & editing. SW: Formal analysis, Supervision, Validation, Visualization, Writing – review & editing.

## Supplementary material

Supplementary Figure 1 The 15 most abundant genera in individual samples.

Supplementary Figure 2 Heatmap depicting the distribution of abnormal genera in individual samples.
