# Supplementary material for: Exploring the gut mycobiome: differential composition and clinical associations in hypertension, chronic kidney disease, and their comorbidity
Source: Front Immunol. 2023 Dec 14;14:1317809. doi: 10.3389/fimmu.2023.1317809 (PMC10755858; doi:10.3389/fimmu.2023.1317809)
Supplement: Supplementary file 1 [file Table_1.docx]

**Table S1 Associated diseases and functional categories of cytokines**

| Cytokines | Alteration in HTN+CKD | Associated disease (s) | Functional categories |
| --- | --- | --- | --- |
| FGF basic | Increased | CKD[1] | Growth Factor |
| GROα | Increased | SLE[2] | Chemokine |
| IFN-γ | Increased | Spontaneous miscarriage[3] | Interferon |
| IL2Rα | Increased | Acute kidney injury[4] | Interleukin |
| IL4 | Increased | Acute kidney injury[5] | Interleukin |
| IL18 | Increased | Acute kidney injury[6] | Interleukin |
| LIF | Increased | Tubular injury[6] | Cytokine |
| MIF | Increased | Kidney disease[7] | Cytokine |
| SCF | Increased | Kidney disease[8] | Growth factor |
| SDF1α | Increased | Diabetic nephropathy[9] | Chemokine |
| TNF-α | Increased | Hypertension and kidney disease[10] | Tumor Necrosis Factor |
| IL9 | Decreased | CKD[11] | Interleukin |
| TNF-β | Decreased | Intervertebral disc degeneration[12] | Tumor Necrosis Factor |

**References**

1. Bozic M, Betriu A, Bermudez-Lopez M, Ortiz A, Fernandez E, Valdivielso JM. Association of FGF-2 Concentrations with Atheroma Progression in Chronic Kidney Disease Patients. *Clin J Am Soc Nephrol* (2018) **13**: 577-584.

2. Zeng Y, Lin Q, Yu L, Wang X, Lin Y, Zhang Y *et al*. Chemokine CXCL1 as a potential marker of disease activity in systemic lupus erythematosus. *BMC IMMUNOL* (2021) **22**: 82.

3. Micallef A, Grech N, Farrugia F, Schembri-Wismayer P, Calleja-Agius J. The role of interferons in early pregnancy. *GYNECOL ENDOCRINOL* (2014) **30**: 1-6.

4. Cho E, Lee JH, Lim HJ, Oh SW, Jo SK, Cho WY *et al*. Soluble CD25 is increased in patients with sepsis-induced acute kidney injury. *Nephrology (Carlton)* (2014) **19**: 318-324.

5. Zhang MZ, Wang X, Wang Y, Niu A, Wang S, Zou C *et al*. IL-4/IL-13-mediated polarization of renal macrophages/dendritic cells to an M2a phenotype is essential for recovery from acute kidney injury. *KIDNEY INT* (2017) **91**: 375-386.

6. Sirota JC, Walcher A, Faubel S, Jani A, McFann K, Devarajan P *et al*. Urine IL-18, NGAL, IL-8 and serum IL-8 are biomarkers of acute kidney injury following liver transplantation. *BMC NEPHROL* (2013) **14**: 17.

7. Lan HY. Role of macrophage migration inhibition factor in kidney disease. *Nephron Exp Nephrol* (2008) **109**: e79-e83.

8. Zhang W, Jia L, Liu D, Chen L, Wang Q, Song K *et al*. Serum Stem Cell Factor Level Predicts Decline in Kidney Function in Healthy Aging Adults. *J NUTR HEALTH AGING* (2019) **23**: 813-820.

9. Takashima S, Fujita H, Fujishima H, Shimizu T, Sato T, Morii T *et al*. Stromal cell-derived factor-1 is upregulated by dipeptidyl peptidase-4 inhibition and has protective roles in progressive diabetic nephropathy. *KIDNEY INT* (2016) **90**: 783-796.

10. Ramseyer VD, Garvin JL. Tumor necrosis factor-α: regulation of renal function and blood pressure. *Am J Physiol Renal Physiol* (2013) **304**: F1231-F1242.

11. Zhang L, Wu JH, Otto JC, Gurley SB, Hauser ER, Shenoy SK *et al*. Interleukin-9 mediates chronic kidney disease-dependent vein graft disease: a role for mast cells. *CARDIOVASC RES* (2017) **113**: 1551-1559.

12. Guo Z, Qiu C, Mecca C, Zhang Y, Bian J, Wang Y *et al*. Elevated lymphotoxin-α (TNFβ) is associated with intervertebral disc degeneration. *BMC Musculoskelet Disord* (2021) **22**: 77.
