# Supplementary figures and images for: Exploring the gut mycobiome: differential composition and clinical associations in hypertension, chronic kidney disease, and their comorbidity
Source: Front Immunol. 2023 Dec 14;14:1317809. doi: 10.3389/fimmu.2023.1317809 (PMC10755858; doi:10.3389/fimmu.2023.1317809)

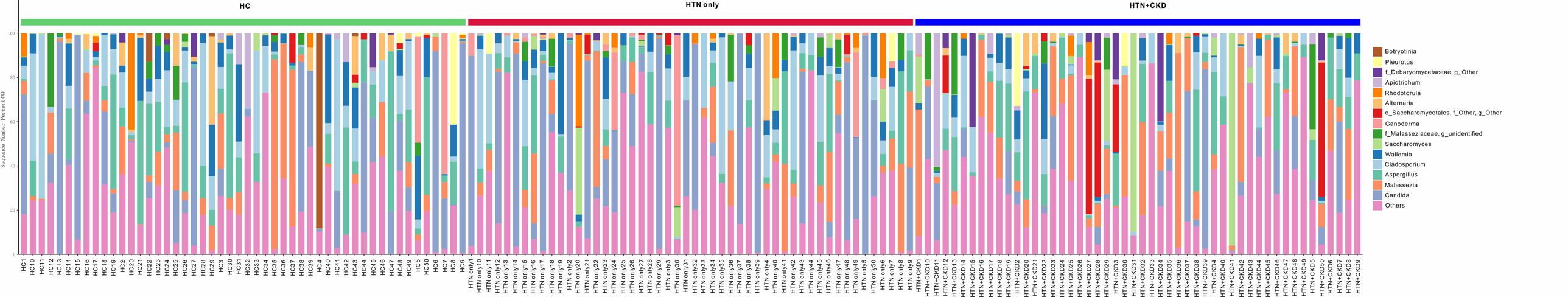

Supplement: Supplementary Figure 1 — The 15 most abundant genera in individual samples. [file Image_1.jpeg]

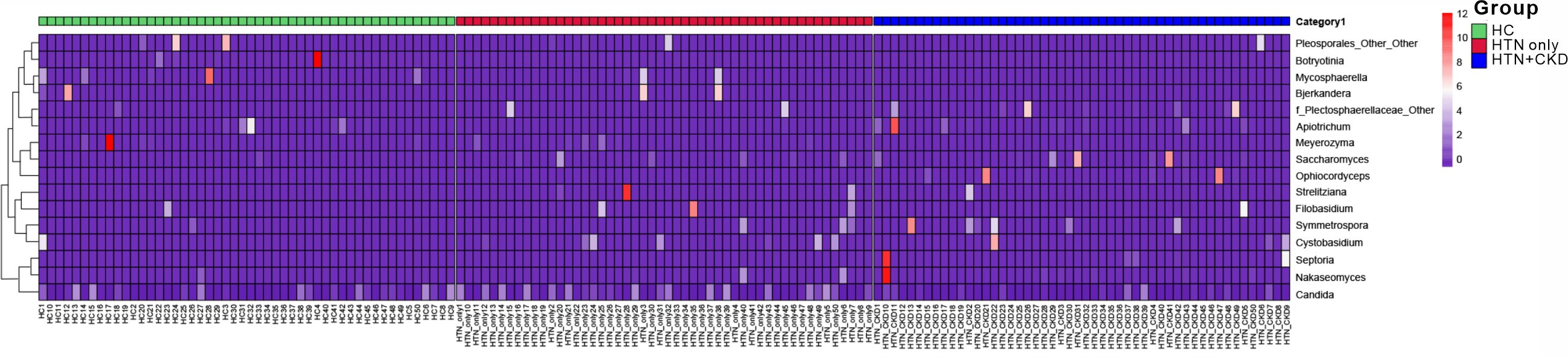

Supplement: Supplementary Figure 2 — Heatmap depicting the distribution of abnormal genera in individual samples. [file Image_2.jpeg]
